# Changes in Foods Served and Meal Costs in Boston Family Child Care Homes after One Year of Implementing the New Child and Adult Care Food Program Nutrition Standards

**DOI:** 10.3390/nu12092817

**Published:** 2020-09-15

**Authors:** Mary Kathryn Poole, Angie L. Cradock, Erica L. Kenney

**Affiliations:** 1 Department of Nutrition, Harvard T.H. Chan School of Public Health, 665 Huntington Avenue, Boston, MA 02115, USA; ekenney@hsph.harvard.edu; 2 Department of Social and Behavioral Sciences, Harvard T.H. Chan School of Public Health, 677 Huntington Avenue, Boston, MA 02115, USA; acradock@hsph.harvard.edu

**Keywords:** child care, CACFP, nutrition policy

## Abstract

This study aimed to determine the impact of 2017 revisions to the Child and Adult Care Food Program (CACFP) nutrition standards on foods and beverages served and meal costs in family child care homes (FCCHs). Our pre–post study utilized four weeks of menus and food receipts from 13 FCCH providers in Boston, MA prior to CACFP nutrition standards changes in 2017 and again one year later, resulting in *n* = 476 menu observation days. We compared daily servings of food and beverage items to the updated standards. Generalized estimating equation models tested for changes in adherence to the standards and meal costs. FCCHs offered more whole grains and less juice and refined grains from baseline to follow-up. FCCHs were more likely to meet the revised whole grain standard at follow-up (OR = 2.7, 95% CI: 1.4, 5.2, *p* = 0.002), but rarely met all selected standards together. Inflation-adjusted meal costs increased for lunch (+$0.27, *p* = 0.001) and afternoon snack (+$0.25, *p* = 0.048). FCCH providers may need assistance with meeting CACFP standards while ensuring that meal costs do not exceed reimbursement rates.

## 1. Introduction

Over 60% of children between the ages of 3 and 5 years in the United States (U.S.) are cared for in early care and education settings [1]. These settings influence the dietary habits of young children since some children may consume up to two-thirds of their daily food intake during program attendance [2,3]. Moreover, early childhood is a critical time when food preferences are established [4]. Early exposure to a nutritious diet may help to prevent excess weight gain and diet-related disease in adulthood [5,6].

The Child and Adult Care Food Program (CACFP), a national program of the U.S. Department of Agriculture (USDA), reimburses child care providers for meals that adhere to the program’s nutrition standards. The Healthy, Hunger-Free Kids Act of 2010 required the USDA to develop new nutrition standards for CACFP to align them with current nutrition science [7,8]. These were designed to be cost-neutral for providers. The updated nutrition standards went into effect on 1 October 2017 and required that participating programs across the U.S.: (1) serve a fruit and vegetable with lunch; (2) limit 100% juice to one serving per day; (3) serve whole grains for at least one grain component; (4) prohibit reimbursement for grain-based desserts; (5) serve yogurt with no more than 23 g of sugar per 6 ounces; (6) serve breakfast cereal with no more than 6 g of sugar per dry ounce; (7) serve unflavored skim or 1% milk; and (8) remove on-site frying [9]. Prior to these standards going into effect, providers could serve a fruit *or* vegetable at lunch, there was no limit on 100% juice, there was no whole grain requirement or prohibition on grain-based desserts, there were no limits on sugar contents of yogurts and cereals, and frying was permitted [10].

Before the effective date of 1 October 2017, studies showed that both child care centers and family child care homes (FCCHs), where providers care for a small number of children in their place of residence, were well-positioned to adopt the new CACFP nutrition standards [11,12]. Among a sample of California child care programs, FCCHs had lower adherence to standards for serving sugary cereals and grain-based desserts compared to centers [12]. However, there is little information about implementation of new nutrition standards in FCCHs that also participate in CACFP. The lack of relevant data on meals provided in FCCHs is important because these settings provide care for over one million children in the U.S., and two-thirds of these children enrolled in FCCHs are provided meals supported through CACFP [13]. However, FCCHs often do not have the same resources and economies of scale as child care centers. They operate with few staff [14] and may have less access and ability to attend trainings [15,16,17] and report challenges with meeting nutrition standards [12], making them important settings for further evaluation. Additionally, documenting potential financial barriers to providing foods that meet the new standards is relevant as prior research suggests that providing healthier meals in FCCHs may cost more [18].

The objectives of this study were to evaluate whether foods and beverages served to 3- to 5-year-olds in FCCHs located in Boston, Massachusetts and the related food costs changed from before to after the new CACFP nutrition standards went into effect on 1 October 2017. We hypothesized that adherence to updated standards would improve and that food costs would increase from baseline to follow-up.

## 2. Materials and Methods

### 2.1. Study Design and Population

This study presents a pre–post analysis of the food environment in CACFP-participating FCCHs in Boston where providers can care for up to 10 children from between August and October 2017 (prior to when the new CACFP nutrition standards were mandatory) to between July and December 2018. Providers were eligible to participate in the study if they (a) operated a licensed family child care program in Boston; (b) served at least one 3- to 5-year-old; and (c) participated in CACFP.

To recruit participants, we downloaded a list of 396 licensed FCCHs with valid contact information from the Massachusetts Department of Early Education and Care website [19]. We first notified providers about the study by a flyer from the Boston Public Health Commission, which had existing relationships with most Boston FCCHs. Providers were selected at random from the list of FCCHs until the target baseline sample size of 30 participants had been reached. Study staff contacted providers by email and phone to verify eligibility criteria and to request participation. Providers that could not be reached after three contact attempts by e-mail and phone were considered nonresponders. We offered providers a $40 gift card as an incentive at both baseline and follow-up. The study was determined nonhuman subjects research by the Harvard T.H. Chan School of Public Health Institutional Review Board.

### 2.2. Measures

#### 2.2.1. FCCH Provider Characteristics

At baseline and follow-up, we collected background information for FCCH providers including primary language spoken by provider (English or Spanish), years of operation, number of enrolled children between the ages of 3 and 5 years, and employment of an assistant(s). We also asked providers about the types of daily meals (i.e., breakfast, morning snack, lunch, afternoon snack, and/or supper) served to children and for which meals they received CACFP reimbursements.

#### 2.2.2. Adherence to New CACFP Nutrition Standards

To assess how consistently FCCH providers adhered to the updated CACFP nutrition standards at baseline and follow-up, we assessed menus for how many days, on average, each provider met the standards for 3- to 5-year-olds related to fruits/vegetables, 100% juice, whole grains, grain-based desserts, yogurt, cereal, and 1% milk. The standard for not frying items on site could not be assessed with menu data and was not evaluated. Research assistants collected at least four weeks of FCCH providers’ written or printed menus used prior to 1 October 2017 and at one-year follow-up. Study staff spoke with providers to collect information on any unclear menu items, particularly types of grain products and milk served and brands of yogurt and cereal. Researchers also verified the brands and types of products in providers’ cupboards, when feasible. Additionally, research assistants trained FCCH providers to photograph children’s plates during meals and snacks to document food and beverages consumed which researchers used to assess agreement between the menu listings and the foods and beverages served at meals and snacks [20].

A total of 1583 meals (*n* = 730 at baseline and *n* = 853 at follow-up) across 476 menu days (*n* = 224 at baseline and *n* = 252 at follow-up) were included in the analysis after excluding 162 meals that were not reimbursed by CACFP. We categorized foods as: fruits (excluding juice); vegetables; whole grains (first ingredient of product is a whole grain); refined grains (first ingredient is not a whole grain); grain products of unknown whole grain content; lean meat or meat alternates (nonfried poultry, fish, or soy product); red meat (beef, pork, lamb, goat); processed meat (bacon, sausage, or other cured meats); cheese; yogurt; eggs; nuts/nut butters; and beans/legumes. We categorized beverages as low-fat or skim milk, reduced-fat milk, whole-fat milk (and further classified as flavored/unflavored), water, 100% juice, or sugar-sweetened beverages (SSBs). Cookies, sweet pie crusts, doughnuts, cereal bars, breakfast bars, granola bars, sweet rolls, toaster pastries, cake, and brownies were classified as grain-based desserts, in keeping with the CACFP definition [9]; sweet crackers (e.g., animal crackers) were examined independently since an exception was granted for these crackers shortly before the rule went into effect [21]. We calculated yogurt and cereal sugar content using nutrition information from manufacturers’ websites.

We coded the number of servings per food or beverage category assuming the minimum CACFP standard serving size (e.g., cups, ounces) for 3- to 5-year-olds [22] in each meal or snack. Then, we created a menu score out of five possible points by scoring one point for each daily serving standard met for fruits/vegetables, whole grains, grain-based desserts, milk, and juice. Standards for yogurt and cereal were excluded since these only applied to a small subset of days when these items were served. We then summed the servings by meal and day to compare to the five selected standards.

#### 2.2.3. Costs

We requested food receipts from grocery store purchases for the same weeks as the collected menus from FCCH providers. Receipts were available for 27.3% of food and beverage items at baseline and 22.9% of items at follow-up. Researchers estimated the number of servings per item purchased based on CACFP standards by meal for 3- to 5-year-olds [22]. For example, if an FCCH provider served spaghetti and meat sauce for lunch, we located the ingredients (i.e., spaghetti noodles, sauce, and meat) on the corresponding food receipt. If the FCCH provider purchased two packages of spaghetti noodles (16 ounces each), we estimated this amount to be equivalent to about 30 CACFP servings (1 serving = 1/4 cup of cooked pasta). Next, we computed the cost per serving of each food or beverage by dividing the cost listed on the receipt by the total number of servings per package purchased. Using the same example as above, we divided the total cost of the pasta purchase ($1.98) by 30 servings. We then repeated this process for the meal’s remaining ingredients (pasta sauce and meat) using appropriate CACFP serving sizes and summed to calculate an average cost per serving of that menu item.

When food receipts were not available, study staff converted the CACFP serving size to grams for each menu item with the USDA’s National Nutrient Database for Standard Reference which contains nutrition information for over 8000 foods [23]. For example, if an FCCH provider served a grilled cheese sandwich for lunch, we estimated the gram weight per CACFP serving of bread (1 slice) and cheese (1.5 ounces). Cost per serving was estimated using an established method [24,25] of matching foods to the USDA Center for Nutrition Policy and Promotion (CNPP) price database [26,27] which contains the average national price per 100 g in 2003–2004 for over 4000 foods. Using the example above, we then estimated the cost per serving of bread and cheese to find a total cost per grilled cheese sandwich.

Costs were adjusted for inflation to 2018 dollars using the Consumer Price Index for All Urban Consumers from the Bureau of Labor Statistics [28]. After combining the number of servings and food costs of the food and beverages from receipt and nonreceipt data, we summed costs per food or beverage category across CACFP-reimbursed meals and snacks.

### 2.3. Analysis

To assess adherence to the updated CACFP nutrition standards at baseline and follow-up, we created binary variables for meeting each CACFP nutrition standard (“no” = 0, “yes” = 1) and all daily CACFP nutrition standards (“no” = 0, “yes” = 1). We then calculated an average menu score across FCCH providers for baseline and follow-up as well as average inflation-adjusted costs per meal. Descriptive statistics were used to summarize the number of servings per food or beverage category served at each meal and snack. We used PROC SURVEYMEANS and PROC SURVEYFREQ in SAS Version 9.4 (Cary, NC, USA) [29] to adjust for the clustering of repeated observation days within FCCH providers.

Generalized estimating equation (GEE) models, which accounted for the clustering of menu observation days within providers, compared the number of days each of the CACFP nutrition standards and the number of days all of the CACFP nutrition standards were met at baseline and follow-up. GEE models also compared the average number of servings per food or beverage category per meal, average menu scores, and the average food and beverage costs per meal. We used PROC GENMOD in SAS Version 9.4 (Cary, NC, USA) [29].

## 3. Results

Twenty-nine FCCH providers, representing 11% of those contacted by our team and 7% of all Boston FCCH providers, agreed to participate in the study at baseline. Nearly half (47%) of those contacted declined to participate due to busy schedules or lack of interest, and 97 (37%) could not be contacted and were marked as nonresponders. An additional 56 (21%) were ineligible for the study (37 did not have 3- to 5-year-olds enrolled and 19 providers did not participate in CACFP). Of the 29 FCCH providers who participated in baseline data collection, 13 remained at one-year follow-up. Reasons for loss to follow-up included eight (50%) FCCH providers citing lack of time, four (25%) FCCH providers closing their program, and four (25%) no longer having 3- to 5-year-olds enrolled. FCCH providers lost to follow-up did not vary by program size or types of meals served, but they were more likely to report speaking Spanish as their primary language.

### 3.1. FCCH Provider Characteristics

FCCH providers reported having an average of three children (SD: 2) between the ages of 3 and 5 years in their child care program at baseline and four children (SD: 2) in this age group at follow-up (Table 1). Most (77%) reported English as their primary language and fewer (23%) reported Spanish. All FCCH providers reported serving lunch and afternoon snack, most (92%) served breakfast, a majority (69%) served morning snack, and less than half (47%) served supper.

### 3.2. Adherence to New CACFP Nutrition Standards

Across 476 total menu days at both baseline and follow-up, we analyzed an average of 62.5 (SD: 20.6) meals per FCCH provider at baseline and 71.8 (SD: 35.5) meals per FCCH provider at follow-up. For CACFP-reimbursed meals and snacks, FCCH providers served an average of 17.2 (SD: 5.1) lunches, 15 (SD: 8.2) breakfasts, 15.2 (SD: 6.7) afternoon snacks, 4.1 (SD: 7.8) morning snacks, and 4.7 (SD: 6.9) suppers at baseline, and an average of 19.3 (SD: 8.3) lunches, 16.4 (SD: 11) breakfasts, 15.5 (SD: 10.6) afternoon snacks, 7.8 (SD: 10.8) morning snacks, and 6.5 (SD: 8.9) suppers at follow-up. At baseline, 75% of menu items matched the meals in FCCH provider photographs.

At baseline, FCCH providers provided meals and snacks that were fairly consistent with pending CACFP nutrition standards on most menu days prior to their going into effect (Table 2), though less frequently served a fruit and vegetable at lunch (50.2% of menu days) or whole grains at least once a day (36.2% of menu days). At follow-up, FCCH providers were more likely to adhere to some but not all nutrition standards as hypothesized. FCCH providers were more likely to meet the standard for whole grains on menu days compared to baseline (adjusted OR = 2.7, 95% CI:1.4, 5.2, *p* = 0.002), but did not significantly change their adherence to other standards. The average daily menu score, which measured adherence to five of the standards, was 3.57 (SE: 0.14) at baseline and rose to 4.02 (SE: 0.09) at follow-up (*p* = 0.01). The proportion of menu days where all daily standards were met increased from 10.3% of menu days at baseline to 30.2% of menu days at follow-up, but this change did not reach statistical significance.

**Figure 1:**
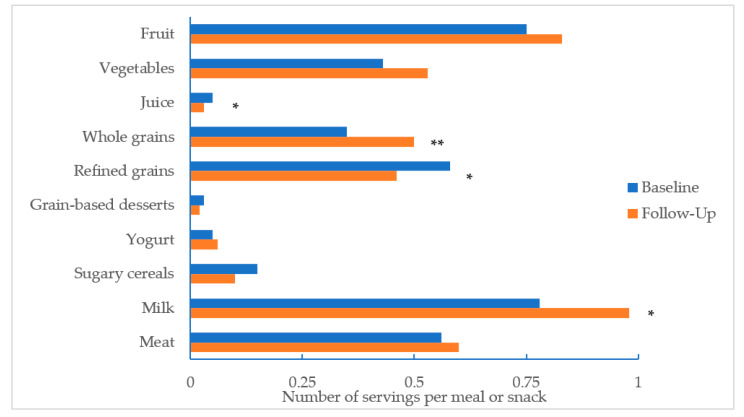
Average number of servings by food category per meal or snack, before and after implementation of new CACFP nutrition standards (*n* = 1583 meals across 476 menu days). Note: * *p* < 0.05 ** *p* < 0.01.

Across the 1583 meals or snacks, the average number of servings of 100% juice decreased (−0.02 servings, *p* = 0.02) and the average number of servings of 1% milk increased (+0.2 servings, *p* = 0.03) from baseline to follow-up (Figure 1). The average number of whole grains servings increased from baseline to follow-up (+0.15 servings, *p* = 0.005) and decreased for refined grains (−0.12 servings, *p* = 0.02). Servings per meal of fruits, vegetables, grain-based desserts, sugary yogurts, sugary cereals, and meat/meat alternates did not change significantly.

### 3.3. Costs

**Figure 2:**
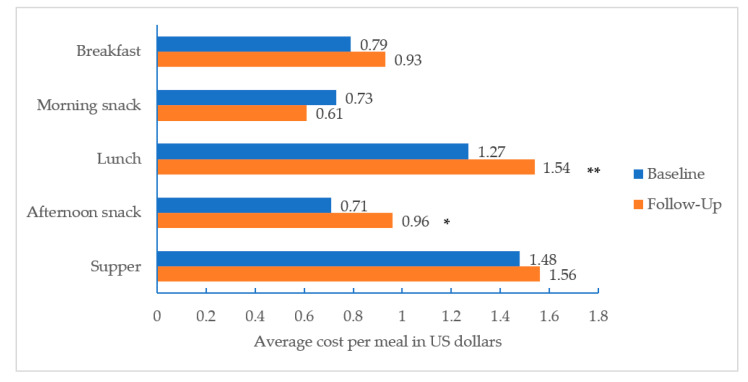
Average cost per meal or snack, before and after implementation of new CACFP nutrition standards (*n* = 1583 meals across 476 menu days). Note: * *p* < 0.05 ** *p* < 0.01.

Average food or beverage inflation-adjusted costs per meal increased from $1.27 to $1.54 for lunch (+$0.27, *p* = 0.001) and from $0.71 to $0.96 for afternoon snack (+$0.25, *p* = 0.048); changes in costs for breakfast, morning snack, and supper did not statistically significantly increase as hypothesized (Figure 2).

## 4. Discussion

This study summarizes the types and costs of foods and beverages served to 3- to 5-year-olds in CACFP-participating FCCHs in Boston before and after the CACFP nutrition standards were modified on 1 October 2017. Overall, FCCH providers were meeting the standards for juice, milk, grain-based desserts, yogurts, and cereals on most days at baseline, with the exception of the standards for whole grains or serving a fruit and vegetable at lunch. Approximately one year after FCCH providers were expected to adopt the new CACFP nutrition standards, providers were nearly three times more likely to meet the whole grains standard compared to baseline. This finding could be explained by observed increases in knowledge of the whole grains standard among the FCCH providers included in our study [30]. Despite these improvements, however, adherence to the nutrition standards did not improve significantly for all of the standards as expected. The whole grains standard was still not met on 38% of menu days at follow-up, and similarly, the standard for serving a fruit and vegetable at lunch was not met on 40% of menu days at follow-up. These findings could be explained by low uptake of training opportunities for the new CACFP nutrition standards among FCCH providers [30]. As we hypothesized, FCCH providers may have experienced higher food acquisition costs for lunch and afternoon snack one year after the new nutrition standards.

This study contributes to the gap in evidence for menu items served in FCCHs and how the 2017 CACFP nutrition standards have impacted the foods and beverages served and costs. Our finding that the standards for juice, milk, grain-based desserts, yogurts, and cereals were met on most days at baseline mirrors most of the findings of two studies [11,12] that documented relatively high existing adherence to nutrition standards prior to mandatory changes within CACFP-participating child care centers. Our results differed from these two studies regarding prechange adherence to the new whole grains standard, which we found to be lower among our sample. This difference could be a product of measurement approaches as we used menu data and pantry reviews to assess adherence with CACFP nutrition standards, whereas the other studies used self-report surveys of practices. The findings that suggest that serving whole grains is an area for improvement, however, are consistent with previous studies that have found the serving of whole grains to be lacking in child care settings [8,31] and confusion among providers about what counts as a whole grain [8,30].

To our knowledge, this study is the first to look at changes for foods and beverages served in CACFP-participating FCCHs from before to after the new CACFP nutrition standards went into effect. Our findings suggest that after one year of implementation, the standards for whole grains and serving a fruit and a vegetable at lunch were still not met on many days. While studies assessing readiness to the meet the new CACFP nutrition standards have found mixed levels of preparedness in child care settings [11,12,31], our results indicate that FCCHs may still not be meeting certain nutrition standards. Training and technical assistance opportunities, particularly for whole grains and serving a fruit and vegetable at lunch, may be beneficial for FCCHs.

Our analysis of meal costs also fills an evidence gap for FCCHs. Although the new CACFP nutrition standards were meant to be cost-neutral, limited data exist documenting whether this actually was the case for participating providers. Our finding that for these providers, lunch and snack costs increased from before to one year after the CACFP nutrition standards were implemented is of concern given that reimbursement rates for FCCHs stayed flat over the same time period [32,33]. Additionally, a previous study suggests that increased costs may serve as a barrier for FCCHs in providing nutritious meals [18]. While our estimates of average costs per lunch and supper suggest these meals could be mostly covered by CACFP reimbursements for low- and high-income providers based on national averages [33], our estimates of average costs per breakfast and snack suggest that the reimbursements may be well below providers’ expenditures, particularly those designated as higher income. For example, FCCH providers in our sample spent an average of $0.96 on afternoon snack, yet average reimbursement rates for high-income providers in the continental U.S. were $0.20 per snack during our study period [33]. In the absence of changes to CACFP reimbursement structures, solutions for budget-conscious shopping lists or sample menus that meet these nutrition standards within the reimbursement structures may be useful tools for supporting FCCHs. Though the meal component responsible for the observed increase in food costs is unknown, it is possible that offering whole grains could partially explain the change in costs. We conducted an exploratory search of a Boston grocery store where many FCCH providers shopped and found a loaf of store-brand whole wheat bread to cost $1 more than store-brand white bread [34]. Connecting FCCH providers to the USDA’s CACFP meal pattern worksheets featuring education topics like grain-based desserts, whole grain criteria, and tips for serving whole grains may be particularly helpful for FCCHs [35].

When new policies are introduced, it is important to ensure that they are adopted as intended and to address potential barriers to implementation. Our study illustrates how well FCCHs were implementing the new CACFP nutrition standards and how meal costs may be a possible challenge for FCCHs. Our study, however, is not without limitations. We lack data from FCCHs that do not participate in CACFP. Generalizability of study findings is also limited due to our study’s low response rate and geographically concentrated, small sample at baseline and follow-up. Additionally, Spanish-speaking providers were more likely to be lost to follow-up. Based on these recruitment challenges, future studies should consider sampling from multiple locales and oversampling for Spanish-speaking FCCH providers.

This study analyzed provider menus and receipts for food purchases. Researchers verified the menu items served by reviewing products in providers’ cupboards where possible, and by comparing photographs of meals to the menus in the subsample of menu days where both photographs and menus were available at baseline finding that the majority of observed matched menus (75%). It is possible that relying on menus resulted in over- or underestimates of the frequency with which menu items were served [36]; however, a previous study found menus to match with 94 to 100% of observed items after accounting for acceptable substitutions within a meal component [31]. Using a direct observation protocol with trained observers may provide a higher quality measure of meal components served, however, consultation with community advisors prior to the project suggested that many FCCH providers are uncomfortable with observers in their home. Using food receipts for actual purchases provides measures of experienced food costs. However, receipts were not available for most menu days and thus we estimated these costs for these days based on national averages in the CNPP price database [24,26,27]. In a recent analysis of improved meal quality in child care settings [25], however, food and beverage items from the CNPP price database were compared to estimates from a local grocery store provider and were found to be within 15% above or below each other. Though this variation in price may contribute to some error, it is not systematically higher or lower by source and is expected to occur across baseline and follow-up data; thus, this method was deemed suitable for estimating food costs.

## 5. Conclusions

One year following the implementation of the updated CACFP standards, Boston FCCH providers served more servings per meal of whole grains and 1% milk and fewer servings of 100% juice and refined grains. However, many providers did not fully meet the standards for serving whole grains at least once per day and serving a fruit and a vegetable at lunch. Food costs for lunch and afternoon snack increased for FCCH providers, which could hinder full implementation of the new standards. FCCHs may need support in meeting the CACFP nutrition standards to ensure that children are receiving nutritious meals.

## Figures and Tables

**Table 1:** Characteristics of CACFP-participating Boston FCCHs included in study, *n* (%).

Characteristic	Baseline (*n* = 13)	Follow-up (*n* = 13)
Program size (number of children enrolled), M(SD) ^1^	8 (2)	8 (2)
Number of 3- to 5-year-old children enrolled, M(SD)	3 (2) ^2^	4 (2) ^2^
Years program has been in operation, M(SD)	4 (1)	4 (1)
FCCH provider employs one or more assistants	9 (69%)	8 (62%)
Primary language spoken by FCCH provider		
English	10 (77%)	10 (77%)
Spanish	3 (23%)	3 (23%)
Types of meals served		
Breakfast	12 (92%)	12 (92%)
Lunch	13 (100%)	13 (100%)
Morning snack	9 (69%)	8 (62%)
Afternoon snack	13 (100%)	13 (100%)
Supper	7 (54%)	7 (54%)

Note: ^1^ M(SD): mean (standard deviation); ^2^ *n* = 12: One FCCH provider did not respond to this survey question; FCCHs: Family Child Care Home; CACFP: Child and Adult Care Food Program.

**Table 2:** Menu days on which Boston FCCHs met selected new CACFP nutrition standards before and after implementation (*n* = 476 menu days from 13 FCCH providers).

CACFP Nutrition Standard	Baseline (*n* = 224 Days)	Follow-up (*n* = 252 Days)	OR (95%CI) ^1^	*p*-Value
Meeting all daily standards ^2^	23 (10.3%)	76 (30.2%)	3 (0.6, 14.3)	0.17
A fruit and a vegetable are served at lunch	112 (50.2%)	150 (59.8%)	1.2 (0.6, 2.5)	0.67
100% juice is served only once per day	223 (99.6%)	252 (100%)	N/A	N/A
A whole grain is served at least once per day	**81 (36.2%)**	**157 (62.3%)**	**2.7 (1.4, 5.2)**	**0.002**
Grain-based desserts are not served	202 (90.2%)	232 (92.1%)	1.2 (0.4, 3.6)	0.69
Yogurt has no more than 23 g of sugar per 6 ounces (among days where yogurt was served, *n* = 38 at baseline, *n* = 58 at follow-up)	38 (100%)	54 (93.1%)	0.9 (0.7, 1.1)	0.30
Cereal has no more than 6 g of sugar per ounce (among days where cereal was served, *n* = 105 at baseline, *n* = 86 at follow-up)	99 (94.3%)	79 (91.9%)	0.6 (0.2, 1.5)	0.28
Unflavored 1% or skim milk for children over two years old	182 (81.3%)	237 (94.1%)	3.7 (0.6, 24.8)	0.17

Note: ^1^ OR (95%CI): odds ratio (95% confidence interval) of new CACFP standard being met per day at follow-up vs. baseline; OR not applicable for juice standard since it was met at both time points; ^2^ includes the following standards: a fruit and a vegetable are served at lunch; 100% juice is served only once per day; a whole grain is served at least once per day; grain-based desserts are not served; unflavored 1% or skim milk for children over two years old. The bold indicate that this finding is statistically significant.
